# Ethnographic closeness: methodological reflections on the interplay of engagement and detachment in immersive ethnographic research

**DOI:** 10.1111/1467-9655.14007

**Published:** 2023-08-17

**Authors:** Caitlin Pilbeam, Trisha Greenhalgh, Caroline M. Potter

**Affiliations:** ^1^ Australian National University; ^2^ University of Oxford

## Abstract

With the reflexive turn in the social sciences, emotional engagement is an inevitable and crucial part of data‐gathering and analysis. However, there is a glaring gap in methodological discussions to this end. Presenting ethnographic research into end of life with people living at home in England with heart failure, we argue for a methodological blend of engagement and detachment that shifts throughout the research process, and that sensory experience is a core part of engagement. We offer ethnographic examples which present and explore some alternatives to emotional engagement and objective detachment: (1) moving with participants to facilitate engagement during fieldwork through shared sensory experience; (2) detachment as a different way of relating when exiting the field and drawing participant relationships to a close; and (3) ethnographic closeness as the interplay of engagement and detachment in participant debriefing and data analysis. Based on well‐established anthropological concepts, and taking both engagement and detachment as embodied and relational, we develop a notion of ethnographic closeness in which detachment is a necessary part. Our detailed methodological discussion thus offers theoretically grounded possibilities and alternatives for approaching and managing the core tension of ‘how close is too close?’ in ethnographic practice. Further contributions supporting researchers in navigating ethnographic research are needed.

## Abstrait

Proximité ethnographique : réflexions méthodologiques sur l'interaction entre engagement et détachement dans la recherche ethnographique en immersion

Résumé

Composante inévitable et essentielle de la collecte et de l'analyse des données depuis le tournant réflexif pris par les sciences sociales, l'engagement émotionnel est pourtant remarquablement absent des discussions méthodologiques. En présentant leurs recherches ethnographiques sur la fin de vie à domicile de personnes insuffisantes cardiaques en Angleterre, les autrices plaident en faveur d'une méthodologie combinant engagement et détachement, dont l’équilibre est amené à changer au long du processus de recherche, et avancent que l'expérience sensorielle est une composante centrale de l'engagement. Plusieurs exemples ethnographiques décrivent et explorent quelques possibilités autres que l'engagement émotionnel et le détachement objectif : (1) bouger avec les sujets de l'enquête ethnographique, afin de faciliter l'engagement par une expérience sensorielle partagée, (2) utiliser le détachement comme un mode relationnel différent au moment de quitter le terrain et de mettre fin à la relation d'enquête (3) pratiquer une proximité ethnographique combinant engagement et détachement dans l'analyse des données et la restitution. Sur la base de concepts anthropologiques bien établis, en considérant aussi bien l'engagement que le détachement comme incarnés et relationnels, les autrices élaborent une notion de la proximité ethnographique dont le détachement est un élément nécessaire. La discussion méthodologique détaillée offre donc d'autres possibilités, fondées sur la théorie, pour approcher et gérer la tension à la limite entre « près » et « trop près », centrale dans la pratique ethnographique. D'autres contributions sont nécessaires pour aider les chercheurs à trouver leur chemin dans la recherche ethnographique.

## Emotional engagement in ethnographic research

With the reflexive turn in anthropology and other social sciences came a focus on the researchers themselves, including anthropologists taking critical account of their own emotions rather than only those of their participants (Bloor & Wood 2011; Evans, McCarthy, Bowlby, Wouango & Kébé 2017; Foley 2002; Hockey 2007; Hubbard, Backett‐Milburn & Kemmer 2001; Venkatesh 2013; Woodthorpe 2011). Anthropological scholarship discussing emotional dimensions of fieldwork (emerging from the 1980s onwards and taking cues from feminist theories) has emphasized that these aspects are often underreported and are therefore an ‘untapped source of insight’, which can be translated and ‘communicated through anthropological reflection’ (Davies 2010: 10). Historically situated in arguing against postmodernist and ‘traditional empirical’ traditions which did not see subjectivity as methodologically useful, emotions were established as epistemologically relevant in participatory research: that is, an important and generative part of knowledge production and understanding (Behar 1996; Davies 2010: 3; Evans *et al.* 2017).

This literature thus explores how emotional experiences can be considered as data, as they are an integral part of how ethnographers relate to participants, make sense and knowledge, and develop representations of those fieldwork experiences. A prominent example highlighting the value of attending to the emotional is given by Renato Rosaldo in his seminal work ‘Grief and a headhunter's rage’ (2017). Rosaldo reflects on the ‘emotional force of death’, discussing the role of grief and rage in elucidating headhunting practices after the untimely death of his wife in the field. Rosaldo discusses how, prior to this experience, he simply had not understood headhunting, despite thirty months of immersive fieldwork over fourteen years. He argues that ethnographies which ‘eliminate intense emotions’, for example by focusing only on the ceremonial or structural, ‘not only distort their descriptions but also remove potentially key variables from their explanations’ (Rosaldo 2017: 161).

Emotional engagement is thus advocated as an essential and inevitable part of the ethnographic research process, from fieldwork to analysis to presentation of findings (Behar 1996; Davies 2010; Jackson 2013: 312; Kleinman & Copp 1993; Stodulka, Dinkelaker & Thajib 2019). In ethnography as an immersive relational practice, emotional dimensions have epistemic value: that is, in developing relationships, understandings, and knowledge (Lo Bosco 2021: 13). However, many accounts available in literature where researchers leverage emotional engagement in analytical methods remain confessional in style (e.g. Rosaldo 2017) and/or predominantly focused on being ‘in the field’ (e.g. Davies & Spencer 2010). These therefore do not explicitly engage with how emotional engagement may be safely and productively handled in practice, and throughout the whole research process. There is similarly little explicit elaboration of other kinds of engagement beyond the emotional, in particular the sensory.

To develop the nature of engagement itself, here we explore how sensory engagement also provides potential routes to ethnographic closeness (Davis & Breede 2015; Myerhoff 1980; Woodthorpe 2011). We draw on our own experiences of ethnographic research at the end of life to critically reflect on the role and limits of emotional engagement in ethnographic practice. End of life is a particularly salient example through which to do this. It exemplifies an ‘intimate and sensitive topic’ where authors have argued that its seemingly ‘universal reach’ has the potential to affect researchers in often profound and personal ways (Borgstrom & Ellis 2021; Woodthorpe 2011: 100); the emotional content of this research is therefore often foregrounded (Borgstrom & Ellis 2017; Brennan & Letherby 2017; Hockey 2007; Johnson & Clarke 2003; Visser 2017). Authors argue that this may be particularly important because ‘unless emotion … is acknowledged’, researchers will be ‘left vulnerable’ and ‘our understandings of the world will remain impoverished’ (Hubbard *et al.* 2001: 119; see also Brannen 1988; Valentine 2007). We see these characteristics and considerations extending beyond end‐of‐life or nominally ‘sensitive’ research areas. Rather than merely ‘sensitive topics’, we identify these aspects as inherent in immersive methods where researchers become *sensitized and sensitive to* their research area, participants, and fieldsites.

We use illustrative ethnographic examples from two years of primary research with heart failure patients in England, conducted by the lead author as part of a Ph.D. and supervised by the co‐authors. This article arose specifically from our ongoing discussions around preparing an early‐career researcher to enter into and subsequently navigate ethnographic end‐of‐life research. The emphasis in ethnographic literature on the importance of emotion left the lead author with a number of key questions that guided discussion: What is the ‘right’ extent of emotional engagement? Do I need to be in this emotionally vulnerable state all the time? What is missing from this perspective? What are other ways of developing meaningful, rigorous ethnographic encounters? How do I safely and ethically *dis*engage from research sites, encounters, and relationships? These questions were further shaped by the context of doing ethnography both in participants’ homes and near researchers’ homes (i.e. the same broad geographical region where the authors lived and worked prior to this study commencing). ‘Entering’ and ‘exiting’ the field were therefore decentred for us as primarily physical acts to do with geography, and led us rather to consider other forms of distance and detachment – aspects we soon realized were an essential part of exploring engagement. These questions highlight gaps and echo calls in ethnographic literatures for methodological transparency, reflexivity, and support (Borgstrom & Ellis 2017: 94; Van Maanen, 2021: 501).

## Forms of detachment and the need for distance

Responding to the narrowing lens of the ‘valorisation of connection, relationship, and engagement’, Yarrow, Candea, Trundle, and Cook argue for tempering this focus on engagement with considering the ‘productive potential of disconnection, distance, and detachment’ (2015: 18, 1). The opposition between detachment and the relational is historically situated in positivist ideologies of ‘objectivity’, but detachment does not have to be considered only in these terms (Trundle 2018). Yarrow *et al.* rather highlight how there are many ways in which detachment and engagement are interwoven, as this relationship ‘is not simple or singular’ but is based on how they ‘limit, complement, and enable each other’ (2015: 1, 3). Influenced by Strathern (1996), they propose that ‘every cut is revealed also as a relation’, and ‘every relation is also a disengagement from something else’ (2015: 24). Detachment, from this perspective, becomes a process of shifting modes of relationality and can therefore be seen as a productive ethnographic practice.

In research contexts where emotions are deemed ‘a crucial and inevitable part’ of the research endeavour (Behar 1996; Brannen 1988; Hubbard *et al.* 2001: 133), a focus on emotions as a manner of engaging with diverse kinds of (inter)subjectivity and (inter)relationality works to combat potentially sanitized accounts (Davies 2010; Evans *et al.* 2017). Anthropologists thus write against such accounts, such as in the case of end of life where the biomedical and life sciences have purview over death and dying (Ariès 1975; Gorer 1965; Green 2008; Hallam, Hockey & Howarth 1999; Kaufman & Morgan 2005; Lawton 2000; Mellor & Shilling 1993; Robben 2017). The critical objective distance advocated by the life sciences is questioned in immersive research, highlighted as an undesirable or even untenable component (Behar 1996; Brennan & Letherby 2017; Hubbard *et al.* 2001; Visser 2017; Woodthorpe 2009; 2011). Ethnographers in different research contexts have highlighted how research may trigger the personal in ways that transgress the boundaries between researcher, self, and participant (Biehl 2013; Brannen 1988; Hockey 2007: 444; Jackson 2013; Maier & Monohan 2010; Valentine 2007: 169; Woodthorpe 2011: 106). However, whilst urging awareness about the emotional labour involved before, during, and after fieldwork, some researchers also highlight the need for some forms of detachment in helping to manage this (Hockey 2007; Koff 2005; Woodthorpe 2011). While in some cases this may include physically removing oneself from geographical fieldsites (as ethnographers have a long history of regional fieldwork), distance can take other forms, which we elaborate below, and do not necessarily correspond to spatial or temporal patterns in linear ways.

In this article, we argue for focusing on the interplay of engagement and detachment, which shifts throughout different stages of the research process, opening up possibilities and alternatives for contextually managing the core tension of ‘how close is too close?' in ethnographic practice. To this end, we further argue that the nature of engagement should be explicitly broadened and elaborated beyond the emotional to include sensory experience as a core element of engagement in ethnographic research (e.g. Davis & Breede 2015; Myerhoff 1980; Woodthorpe 2011). Our discussion of end‐of‐life research is but one example of how the configuration of engagement and detachment might look. We nevertheless offer insights that build on the call for methodological transparency and draw lessons from wider anthropology (Behar 1996; Davies 2010; Yarrow *et al.* 2015). These considerations may be particularly relevant for early‐career researchers with little ethnographic experience, where the nature and extent of engaging and disengaging have important implications for both researchers and participants.

We follow Evans *et al.* in understanding emotions as ‘embodied and relational, existing in‐between people, places, things’ (2017: 586), and Yarrow *et al.* in understanding detachment as neither invoking nor linked to positivist notions of objectivity, but as ‘an embodied practice of detached relationality’ (2015: 2). Within ethnography as a collaborative and intersubjective practice, we similarly take engagement and detachment as ‘interwoven’ (Yarrow *et al.* 2015: 2‐3). Bringing engagement and detachment together into conversation, our ethnographic examples illustrate alternatives to emotional engagement and objective detachment, and how researchers might generate and maintain what we term ‘ethnographic closeness’. This term has been referenced in previous anthropological literature, including discussions of closeness in negotiating boundaries in ethnographic research (e.g. Alvesson 2009; Lombard 2022; Maier & Monahan 2010), yet there has been little consensus on its definition. We therefore define ethnographic closeness here as the methodological blend of engagement and detachment, anchored in the ethnographer's embodied relations to participants and their lifeworlds, involving physical, sensory, and emotional forms of engagement.

## Preparing for and doing ethnographic end‐of‐life research

In this article, we draw and reflect on primary research where the lead author collected data, with ongoing input from co‐authors. References to ‘I’ or ‘me’ therefore denote the lead author's direct engagement with research participants, an account deriving from first‐person fieldnotes. Co‐authors provided significant academic supervision and peer support throughout the research project. This involved ongoing reflexive discussions of the data and lead author's experiences, which particularly focused on theoretical approaches and framings, as well as ethical, methodological, and practical challenges arising. Through these discussions, the lead author became aware of how her own engagement and detachment shifted at different stages of the research process.

Together, we established the strategies discussed here for managing and leveraging this interplay. Through reflecting on what co‐authors felt worked well and less well in previous projects, the process of deciding on the strategies for this study allowed us to explore what ethnographic closeness meant to us and to develop the concept in practice. As co‐authors did not collect ethnographic data in the field for this project, engaging and detaching involved playing an active role in facilitating different aspects of this for the lead author. This included creating space for reflection, debriefing, and methodological decision‐making, and critically encountering the accounts of participants’ lives offered by the lead author. The concept of ethnographic closeness was thus negotiated through this collaboration. References to ‘we’ therefore denote all authors’ engagement with the material, contributing differing theoretical perspectives and expertise, in (medical) anthropology and clinical medicine, to develop the analysis, framing, and interpretation presented in this article.

The research presented stems from a two‐year ethnographic exploration of chronic dying in Oxfordshire, England: people living in their own homes with heart failure towards the end of life. The study was broadly phenomenological, and informed by sensory methods (Ingold 2014; Irving 2017; Pink 2005). The lead author immersed herself in the local landscape of end of life, shadowing Heart Failure Specialist Nurses on home visits, in clinics, and rehabilitation centres; attended carer training, end‐of‐life workshops, and support groups totalling over 130 hours; and spent time going about daily life with fourteen key individuals dying with heart failure, their wider families, and care networks totalling over 200 hours between February 2017 and February 2019. Fieldwork logs giving details of these two phases are included in the appendix.

Heart failure is a chronic, degenerative illness that is not often treated as ‘terminal’, despite having similar five‐year mortality rates to some terminal cancers (Kaufman, Mueller, Ottenberg & Koenig 2011; Klindtworth *et al.* 2015; Steinhauser *et al.* 2011). Those with heart failure experience turbulent, highly uncertain trajectories over time. They may unexpectedly shift from feeling relatively well to experiencing debilitating symptoms including extreme fatigue, breathlessness, and fluid retention. Over periods that can vary from months to many years, medications are up‐titrated and quality of life decreases until (potentially sudden) death (Greenhalgh, A'Court & Shaw 2017; Kaufman *et al.* 2011; Walthall & Floegel 2019).

Whilst preparing for fieldwork with a potentially vulnerable population, the lead author turned to end‐of‐life researchers and literature in order to better equip herself. Considering the perceived or potential vulnerability of participants towards end of life, around which there is some debate (Borgstrom & Ellis 2021; Kendall *et al.* 2007; Lawton 2000; Witham, Beddow & Haigh 2015; Woodthorpe 2011), she took care in being clear and flexible with participants during data collection about obligations to the study. In accordance with the Association of Social Anthropologists’ (2011) guidelines for ethical practice, we therefore sought to be continually mindful of and reflexive about the potentially intrusive nature of ethnographic methods, to honour trusting relationships with participants, and to protect as far as possible the well‐being of all those involved in the research, including ourselves.

From literature, emotions emerged as a construct for dealing with how close one should strive to get to participants. Emotional engagement discussed in the broad terms sketched above left the lead author with little concrete guidance as to how to both practically and ethically build, manage, and withdraw from participant relationships in this context. Whilst equipped with an important awareness of the emotional, her toolkit consisted of striving to be consistently sensitive, engaged, and reflexive. As the study progressed, we became increasingly aware of the important role that detachment was playing in the research in different ways, from physical to sensory to emotional. Facing people who were struggling through illness and life, and towards its end, required dynamic processes throughout research involving both engagement and disengagement, intimacy and detachment.

However, upon immersing herself anew in audio recordings, fieldnotes, and photographs during her analytical journey, the lead author was reminded of the primacy of emotional engagement – and began to worry about the detachment that wove throughout her fieldwork experiences. There was an ever‐looming question, especially present after she had drawn fieldwork to a close: *Did I get close enough?* With an overemphasis on emotion and engagement as beneficial, which seemed to minimize the potential importance of detachment, reflexive processes can move quickly beyond the productive questioning characteristic of ethnographic methods, to becoming destructive of a researcher's own sense of fieldwork experiences and data, recently echoed by Borgstrom and Ellis (2021).

We present ethnographic examples from different stages of this research to develop the notion of ethnographic closeness. These examples explore the relationship between engagement and detachment in terms of the researcher's own shifting relations to participants and their lifeworlds in physical, sensory, and emotional ways, without assuming that engagement and detachment are mutually exclusive. The findings and discussion are structured around: (1) moving with participants to facilitate engagement during fieldwork through shared sensory experience; (2) detachment as a different way of relating when drawing participant relationships to a close; and (3) ethnographic closeness as the interplay of engagement and detachment, in participant debriefing and data analysis.

## Facilitating engagement through sensory experience and movement

The word ‘wiry’ echoed around my head when Nancy^1^ first opened the door of her home to me, and I saw how thin she was. I would have thought her far frailer had she not smiled quite so often, or quite so broadly. Her form was mostly hidden underneath comfortable layers of baggy clothing, her collapsed chest making me want to put a hand on my heart. She welcomed me with a hug that I read as warm in the length of the embrace, when I felt her bones press insistently into mine. Immediately after she had invited me inside, I smelled cigarette smoke. It called to mind what else the GP had related: ‘Still enjoys a fag!’, and what I thought in response: ‘Well, good for her!’ She took me into the kitchen and offered me a cup of tea, proceeding to boil the kettle and flit about the cupboards. I soon realized that Nancy could not sit still for long; it was not so much a sense of restlessness as little sparks of interest that captured her attention and made her dance between conversations, activities, and topics. But her breathing brought on a visceral reaction in me: I could hear her stifling every breath so that it would not come as a gasp, even when she wasn't hacking a wet cough. Every word brought images of raw strands of vocal cords rubbing against each other like sandpaper on silver, destructive. She navigated the discomfort by not speaking too loudly – too loudly would bring on the coughing – and pitched her voice low, using her chest as a resonating chamber where it seemed her voice box had fallen, and was rattling (Excerpt from field diary, 21 February 2018).

Nancy was 84 years old with COPD (Chronic Obstructive Pulmonary Disease) and heart failure, and lived in a small single‐storey house with her husband; she died a few months after I met her. The excerpt illustrates the core ethnographic orientation of this study towards visceral, bodily engagement with participants and fieldsites. Sensory ethnography (rather than ethnography of the senses) is ‘a process of learning through the ethnographer's own multisensory, emplaced experiences’ (Pink 2009: 64) to gather ‘knowledge beyond language’ (Okely 1994: 45; see also Myerhoff 1980) This recalls Csordas's (1993) somatic modes of attention: that is, attending to but also with the body in context. Redefining traditionally psychologized understandings of attention, he suggests rather a bodily ‘turning towards’, constituting ‘bodily and multisensory engagement’ with the ‘body's situation in the world’ (Csordas 1993: 138). Indeed, Davis and Breede advocate combining attention to ‘physical sensations, emotions, contemplation, and dialogue’ in developing a ‘holistic’ ethnography (2015: 79).

Reflecting on the sensoriality of a hug with Nancy thus offers an insight into how I started to build an understanding of and empathy for the way she went about daily life before she died. There was a vast amount of information and sensitivity that I feel I gleaned from those few simple moments when our bodies pressed together, which sharpened my attention to other aspects of her life. The combination of her thin frame protruding into me, the sound of her cough, the feel of her breathing, the smell of cigarette smoke, and the way she held her body and shrouded it intertwined with the narratives of her daily life before and during ill health to become inseparable from the way I encountered Nancy and the data I collected with her.

Whilst emotional engagement was demonstrably part of how I related to Nancy, built a relationship with her, and began to make sense of her life, emotional engagement arose and was supported by other kinds of bodily engagement and attention I cultivated. Gestures that felt affectionate on both our parts, like being led by the hand around her house and garden and noticing how cold her fingers were against mine, shaking slightly; accepting lukewarm mugs of tea with perhaps slightly sour milk; hugging her at every greeting and parting. Through these sensory, bodily, and interrelational activities and aspects, my relationship with Nancy grew and changed partly as a result of the multifaceted closeness we co‐constructed and generated together.

Visits with Nancy and other participants thus involved different kinds of experiences including but not limited to emotional ones. Engaging with participants and their daily lives for me was facilitated and embedded in some kind of movement with them. For participants with changing bodies and unpredictable prognoses, it seems fitting to focus on their processual and dynamic journeys between places, or how they make their way.

I begin to pay attention to the width of the pavement, with different textures signalling bike or pedestrian, and we overtake several people, Rosemary on her mobility scooter (top speed 4 m.p.h.) and me quickening every few steps to keep pace. She is going a bit slower for me than she would have usually, she told me. As we make our way together, I begin to notice anew the texture of the road surface, the closeness and loudness of the cars, the speed of other people, the height of the push button to cross the street and change the traffic lights. We take small detours so that she can go down the sloping ramps built into the pavement to merge with the road, as we ‘go down the shops’ (Excerpt from field diary, 1 November 2017).

Rosemary is 83 years old, a widow who lives alone in her home of thirty years, and manages severe heart failure, spinal spondyloses, and peripheral neuropathy (creeping numbness in her limbs). Her morning routine is elaborate, exhibiting multiple workarounds. Not only does she go shopping six days a week, leaving the house at 8 a.m. sharp every day, but there is a shifting sequence of movements that she goes through before she exits the front door. It was not until l went through these processes with Rosemary that I began to understand what ‘going down the shops’ meant to and for her. Indeed, Ingold and Vergunst (2008: 3) discuss walking with people as a mode of ethnographic enquiry, lamenting how in much ethnographic research the importance and meaning of journeys get lost amongst a focus on destinations.

In moving with participants, I became sensitized to the pathways that they navigate through their lifeworlds. Going to the shops with Rosemary, I had not gleaned from her words the texture of the asphalt, the roughness and unevenness of the surface along which she travelled; nor how it felt to bump along, so much that her voice as she continued to chat to me vibrated with each small hurdle. In short, I had not understood that pathways must be negotiated, and that the easiest‐looking, most direct route is not always an option.

Movement here was about engaging with the back and forth that Rosemary habitually experienced, immersing myself in the context of going about this daily routine together, and cultivating shared experiences. She showed me some of the things she cared about, how and why, and I began to care about them too. She volunteered answers to questions I had not yet thought to ask, such as that this routine now took her almost three hours to complete whilst two years ago it took an hour and a half.

As Pink highlights in her study of English laundry practices and cleanliness, ‘social scientists also benefit from accounting not only for the visual and material aspects of everyday life but also for olfaction, tactile experience, and sound’ (2005: 276). She emphasizes the limitations of relying on visual cues and others’ descriptions in language to access lived experience, though Desjarlais also points out that the sensory is woven like ‘strands of a braided rope’ (2003: 6) into the way people talk about life as lived. In order to unravel some of these strands of significance, it may be necessary not only to emotionally engage with participants but also to do things and be with people as they go about these daily activities. While the emotional engagement that Davies (2010) and others argue for is critical, we elaborate on the nature of engagement and what it might practically entail. The sensory thus sits alongside the emotional, but nonetheless contributes in its own right to how a researcher might tune in, attend, relate to, and reach understandings about participants, their experiences, and lifeworlds.

These sensory practices highlight how moving and engaging bodily with participants became central in my understanding of how to pay attention to not only participants, but also the contexts and environments in which they were embedded and strived to move through. Moving with participants offered a practical starting point to build relationships, share experiences, and generate data together. Embodied engagement, as a shared intersubjective encounter, allowed me to access and report on the journeys and processes of how participants make meaning and sense of their changing lived bodily experiences and shifting everyday practices, in this case with heart failure. This specifically underlines that emotional engagement is not separate from other kinds of engagement with participants and fieldsites. Engagement for me was also sensory and physical: that is, doing things and being with, requiring bodily movement. Emotional engagement is therefore entangled in and facilitated by other kinds of engagement, attention, and experience which may unfold with and through the body. Engagement, however, is not only about intimacy, but also about how one manages and moves away from this. To more fully deal with the many entanglements of engagement, we must consider the importance and role of detachment.

## Detachment as a way of relating

‘The end of my research project is coming up in a few months’, was how I broached the subject towards the end of our conversation, aware that I was still holding the teacup I had drained nearly an hour earlier. Noémie's 19‐year‐old cat was on my lap, and I didn't feel it just to disrupt her after she had spent a good deal of effort to jump up and settle in. Noémie always prepared tea in a silver tea service, with real tea leaves, whenever I came. This was my fifth visit with her, and although we had planned two more, I had decided it was time to start talking with her about our last. ‘So, I won't see you before you go away, and then we'll only have two more visits together before my research project ends. That means that after those two visits, I won't be seeing you again after that, because our research together and data collection will be finished’. I noticed the words I carefully chose, a bit more on the jargon side of academia: I have academic responsibilities, I felt I was telling her, this is not just a ‘normal’ relationship. At the same time, I am also worried about how her trip will go and how she will feel, Noémie expressing concerns about how taxing it could be and the limitations her health might put on her activities in India at age 70. Without missing a beat, Noémie smiled and said: ‘Oh, well, you can always come for tea after the project is finished!’ I didn't know what to say. I don't know if it was ‘the right thing’ to do, but I thanked her for the offer, and told her I would see her when she got back. Then I carefully moved the cat off my lap, in order to leave (Excerpt from field diary, 18 July 2018).

The above excerpt highlights some of the personal, ethical, and practical tensions that doing immersive research brought up for me. Ending research relationships and exiting the field is part of any ethnographic work, yet is less discussed (Jackson 2013; van Maanen 2021). For me, the negotiation of these aspects was foregrounded all the more through the context of end of life. With sudden death a prominent possibility for those managing heart failure, I was aware that any visit with a participant might be the last – regardless of whether I had plans to end the research relationship or not. I also cared about the things that Noémie cared about, and had cultivated what felt like friendship with her and other participants, their families, carers, and even pets. At the same time, I was aware that building close research relationships was part of generating ethnographic understanding and insight. Counter to this immersion and intense engagement, extricating myself from the field and participant relationships seemed to present a dilemma of how to honour these interactions whilst also distancing myself from them.

Drawing research relationships to a close was something that I discussed at length with co‐authors. We decided that the strategy I would use would be a process of distancing and detaching. Detachment had emerged earlier during research as an important strategy whilst doing fieldwork near home, using my own car to travel to and from participants’ homes past which I drove regularly when not visiting them (Pilbeam 2019). In this way, we deliberately framed the fieldsite as participants’ homes, rather than the geographical region itself, allowing us to decentre the notion of ‘exiting the field’ as a primarily physical act of detachment. This opened up detachment as a process, not purely a geographical shift but involving many potential forms of distance.

As the excerpt illustrates, detachment as a process of distancing entailed reintroducing the language of academia and research, repositioning myself in relation to participants as a researcher. Although I felt that I never lost sight of my experiences as fundamentally part of a research project, I framed this as shifting back to ‘researcher mode’. This was not to end engagement altogether, but rather signalled a shift towards a different kind of engagement and relationality with participants as I moved into analysis in earnest. Having worked so hard to establish informal personal relationships, where I would check in regularly with participants and they would let me know when things went wrong (or right), I felt the difficulty of disentangling myself from people who felt more like friends. However, even on a practical level, keeping up with over ten participants was not something I could feasibly continue to do in the same manner indefinitely.

Shifting modes of relationality allowed me to manage expectations of our relationship and encounters whilst still being engaged, in the sense of being attentive and pursuing understandings. This also laid the groundwork for engaging in findings more analytically with participants, as elaborated in the section below. Immersive research may not only ‘blur the perceived or intended boundaries between researcher and self’, but further ‘blur[s] the boundaries between the professional observer/researcher and the person‐behind‐the‐researcher undertaking the research’ (Woodthorpe 2011: 106). Ethnographers highlight that whilst this may generate insights, it may be equally distracting and distressing (Hockey 2014: 98‐100; Valentine 2007: 169‐70; Woodthorpe 2011).

End‐of‐life research is a space where contrasting views of the role of subjectivity in research are particularly visible, given its intersection with the biomedical. For example, Rolls and Relf (2006) suggest a strategy for managing the distracting nature of emotions in their research with childhood bereavement services in the United Kingdom, adapting clinical supervision in the context of qualitative research. They suggest ‘bracketing interviews’ to reduce the impact of researchers’ personal and professional experiences during data collection and analysis. They argued that this increased ‘objectivity and [amplified] the researcher's own reflexive capacity’ whilst holding their own experiences (Rolls & Relf 2006: 286). Contrastingly, in her research in cemetery landscapes in London, Woodthorpe (2011) considers how feasible it is to ever be detached from research in an ‘objective’ sense. She contends that ‘scholarly critical distance’ as objectivity is neither possible nor desirable (Woodthorpe 2011: 99‐100).

These examples echo critiques of scientific objectivity by end‐of‐life researchers and ethnographers more widely, who advocate for embracing the subjective and emotional. But whilst foregrounding engagement is a way of combating accounts of objectivity, the importance of distance is increasingly discussed and recognized (Candea, Cook, Trundle & Yarrow 2015; Maier & Monahan 2010; Trundle 2018). Trundle describes how ‘modes of relational detachment and disconnection’ (2018: 89) are necessary in collaborative fieldwork to respond to emerging practical, ethical, and relational tensions: for example, in response to participants’ requests, when collaborations fail, or when research relationships must be ended. Detachment is thus argued to be a crucial part of ethnography that interacts and is intertwined with daily life, analytical efforts, and ethical standpoints (Yarrow *et al.* 2015). Detachment does not have to equal scientific objectivity.

In our study, relational detachment manifested in how we managed ethical, personal, and practical tensions, such as when shifting the interaction I had with participants. This had very little to do with maximizing objectivity or being objective, in the sense of seeing things without the distraction of strong emotions. I found distancing an odd shift to make, one that required a number of reminders that felt transgressive of the concept of ‘friendship’, and was therefore something that did not preclude examination through emotional engagement as well as other forms of reflexivity. Far from being a ‘clearer’ way to access data, detachment did not necessarily comprise or rely on physical distance from participants and fieldsites, but was a practical strategy for managing participants’ and my own relationships, boundaries, and expectations at particular points in the research process.

Some researchers reference the importance and forms of distancing practices even whilst still ‘in the field’, and even in small ways that separate their more professional research personas from the rest of their lives. Forensic anthropologist Clea Koff (2005) describes how she rotated three bras during her fieldwork when working on mass grave sites in recent genocides in Rwanda and other countries: one for being at the excavation site, keeping it in a plastic bag to contain the indelible smell of decomposition that clung to it; one for wearing after field excursions when she did not have the luxury of a proper shower; and one she wore when she had a ‘sacred’ shower in which she could clean herself more thoroughly.

Whilst we agree that objectivity in the sense of the biomedical and life sciences should not be an aim of immersive ethnography, detachment should be decoupled from objectivity (Candea *et al.* 2015; Trundle 2018) and similar concepts in order to highlight it as a productive ethnographic practice that does not oppose or preclude engagement. Detachment may be an especially important aspect to balance with engagement in immersive research where the emotional and personal impact is seen as unavoidable. Particularly in drawing my research to a close, I found that some kind of detachment was necessary, even desirable, as part of managing, accessing, and reporting on the endings of certain ways of relating to participants and the elaboration of others. Detachment here highlights how this is processual, neither an absolute state nor mutually exclusive with engagement. Rather, processes of detachment open up possibilities for different or unexpected modes of engagement and interrelationality with participants and data, such as in analysis.

## Ethnographic closeness as the interplay of engagement and detachment

Towards the end of fieldwork, as part of drawing fieldwork relationships to a close as well as recapturing closeness in different ways, we wanted to feed back to participants some emerging findings. We hoped to give them an insight into the project as a whole whilst progressing, and offer them the opportunity to feed back on whether the written text resonated with them, or needed amending. This was done in the vein of ‘member checks’: for example, checking interview transcripts and interpretations with participants to ensure they reflect and resonate with their experiences. The strategy we decided on was a personalized ‘thank you’ card, enclosing an excerpt of the ethnographic vignettes produced through the research and a summary of findings with my contact details. For example:

Dear Kent and Carolyn,

Over the course of my research, I was privileged enough to have been invited into the homes of 14 people, all of whom have expressed diverse hopes and worries, triumphs and challenges. Hoping to show clinicians how rich and insightful anthropological research can be, I wrote illustrative ‘vignettes’ of all the people I have met. An edited excerpt of what I have written about you is given below (I have used pseudonyms to protect your confidentiality).

*Each time I visit Kent and Carolyn, Carolyn greets me warmly and leads me through the long house to Kent. He is sitting in an adjustable chair, where he tells me he spends most of his life. Over months, I have begun to understand how the couple's lives intertwine whilst also moving separately to one another, often in complementary ways. I have witnessed a positive upturn in both Kent's health and his outlook on life, as his gout‐afflicted feet are healing, and he feels that life is more worth living*.

Although everyone has their own unique stories and experiences, I also wanted to share with you some of the more common themes that I am writing about in my final thesis. Not every theme may apply specifically to you, but hopefully some of them resonate with your experience.

This excerpt was followed by a generic summary of shared themes. Prior to sending these letters, I told participants to expect them and that I would call or have a final visit where we could discuss them. This served the dual purpose of allowing findings to be more co‐constructed, generating further interrelational experiences and also of concretely returning me to the role of ‘researcher’. My very first participant was also the last person I visited, and she called some of her family to come to say goodbye, people I had met and also spent time with over the course of two years.

Throughout this process, I became more acutely aware of how anthropological framings may differ from conventional accepted narratives; in this case, that heart failure was not at that time habitually communicated to patients as a terminal illness (Barclay, Momen, Case‐Upton, Kugn & Smith 2011; Exley 2004; Hjelmfors, Strömberg, Friedrichsen, Mårtensson & Jaarsma 2014; Parry, Land & Seymour 2014). During the research, I had been mindful of not suggesting to participants that they were ‘dying’ when they might not consider themselves to be so. As the explicit framing of the project aimed to reintroduce the ‘living into dying’, we utilized this focus on living to introduce the project to participants. This allowed people to tell me about what was important in their lives, dying arising organically in conversations in a participant‐led manner over the course of our relationship, rather than pursued as a theme from inception. However, in feeding back findings to participants as a whole, we could not entirely rely on the original framing, as dying and end of life were indeed an undertone to many of the themes we wished to reflect.

Aware that this and other aspects of the research would not resonate with every individual amongst participants, I neither emphasized nor shied away from dying as a topic. In inviting participants’ feedback, I built in reminders that the aims of the project – and anthropology in general – was to learn from diversity in meaningful ways, and therefore perhaps not every theme would be relevant to their lives. These considerations highlight the intertwined processes of engaging and detaching that I shifted between and balanced during reflection and analysis.

The task of synthesizing brief summaries with the specific audience in mind, of those who were closely involved in our research, was a key part of my analytical process, illustrating the productive interplay of engagement and detachment. As an exercise that was part of a distancing practice, this in fact facilitated close examination of how I had dealt with and pursued key themes of my research as my relationships with participants grew and changed. After drawing fieldwork to a close and no longer being ‘on site’, this was also a useful way of re‐immersing myself in these experiences and impressions in a contained, achievable way that moved my analysis forward. Forcing me to critically encounter how participants themselves would find reading these findings, the interaction of engagement and detachment gave rise to different modes of interaction and reflection.

Writing of the ‘social life of interview material’ in end‐of‐life research, Hockey (2014) describes the complex negotiation of these fragments that bear pieces of people who have died, such as audio recordings. In analysis, an ongoing iterative process that came in and out of focus, the summary I produced for participants became a sounding board, similarly growing and changing. This maintained my sense that data were co‐created and attached to a person, through handling, experiencing, and attending to them in different ways to generate insights.

This echoes Yarrow *et al.*’s (2015) assertion that engagement and detachment extend and enable each other, as well as Trundle's discussion of how ‘everyday fieldwork collaborations … require careful oscillation between modes of distance and intimacy’ (2018: 90). In seeking to achieve ethnographic closeness as a route to understandings of participants, their lifeworlds, and experiences, this necessarily involves a methodological blend of engagement and detachment that shifts appropriately and responsively throughout different stages of the research process.

Whilst I have been going over the same material, considering it anew and continuing to write about it, hearing participants’ voices on audio‐recordings or seeing their faces in memories and illustrations, I do wonder what and if ever participants think of me. I was delighted, and touched, to have received an email from Emily a few months before completing my thesis. Interested in her further impressions, but also how she continued to give me an update much as she would in person, I include it below, unedited/unformatted, sent 17 April 2019.

Hello Caitlin at last I have remembered to ask one of the family to show me how to get your name into my iPad, never too old to learn !! Do hope all goes well for you, thankyou for your letter , your studies have all been varied from so many people , we are all a bit different for sure , I expect through the years different memories will go through your mind as you continue with your work , I'm still pottering on , had a few set backs with the cold bug , then. “Old age ” got into my back for couple of weeks , but it was kidney infection,so antibiotics seem to have settled it , I'm still looking for the energy pills to give me a bit more “ go” !! I wish you all the best as you continue with your career , enjoy all life has to offer you, it all slips away so fast, I cannot believe, if all's well I will be 90  in August , my head still thinks I'm younger , I wish you a Happy Easter , hoping too we have a dry sunny weekend , take care of yourself, fond memories from Emily x

Sent from my iPad

Despite the fact that I had not been physically in the field for months, this email thrust me back into a kind of intimacy with Emily and the data collected with her, which I afterwards encountered subtly anew in an ongoing reflexive process of contextualizing, challenging, and sense‐making. Out of distancing practices that leave space for the uncertainty, plurality, and incompleteness of ethnographic research, unexpected opportunities for diverse kinds of intimacy and (re‐)engagement may thus arise. Given the open‐ended nature of ethnographic enquiry, this and other examples – the smell on Koff's bra, the sound of someone's voice on a recording, an unexpected contact – highlight how neither engagement nor detachment necessarily depends on being physically near or far from participants or fieldsites.

Our notion of ethnographic closeness thus takes the relationship between engagement and detachment as a duality (i.e. both/and), rather than a dualism (i.e. either/or), ‘continuously shaping and being shaped by situated practice’ (Schultze & Stabell 2004: 554), which foregrounds its emergent, cyclical, and pragmatic nature. Engagement and detachment are therefore not mutually exclusive, but mutually facilitative and constitutive as part of the process of fieldwork and analysis. Ethnographic closeness, resulting from this dynamic interplay between engagement and detachment, allows ethnographers to access and report on changing relationships with participants, how participants understand their changing relationships with the ethnographer, and how the ethnographer manages these changes in relation to the key themes and findings as the project progresses.

## Conclusions

The researcher's own state of being, reactions, and emotions during ethnographic fieldwork came to be seen as an important part of generating understanding when arguing against scientific objectivity (Behar 1996; Davies 2010; Trundle 2018; Yarrow *et al.* 2015). However, with little detailed methodological discussion of how and to what extent to engage and disengage, particularly beyond being ‘in the field’, ethnographic researchers have few practical tools to approach research which can be so personally and professionally challenging. This leaves less experienced ethnographers in particular potentially unprepared to manage questions of ‘how close is too close?’ (Maier & Monahan 2010), as we illustrated through describing our own research process.

Drawing on the increasing recognition of the need for detachment as an essential part of the research process (Candea *et al.* 2015; Trundle 2018), we have taken both engagement and detachment to be embodied and relational, and considered them together to enable us to explore their interplay at different research stages in generating what we term ethnographic closeness. Through probing ethnographic closeness, we explicate the relationship between engagement and detachment as dynamic and embedded in situated practice, and question whether heightened states of emotional engagement are always appropriate. We contribute to the anthropological literature by expanding on the nature of engagement to frame and argue for sensory experience as a core aspect; and demonstrating detachment as the methodological companion to engagement in our delineation of ethnographic closeness. Through ethnographic examples, we offered a transparent methodological discussion illustrating some practical alternatives to full‐on emotional engagement throughout the research process.

Whilst the level and kind of engagement must be appropriate to the researcher, participants, context, and research stage, considering complementary forms of engagement such as the sensory does not preclude the emotional. Drawing on sensory ethnography as a mode of bodily attentiveness in building awareness, understanding, and relationships (Csordas 1993; Pink 2005; Ingold 2014) opens up practical strategies like doing things, being, and moving with participants to offer productive routes to ethnographic closeness. Further, generating ethnographic closeness necessarily entails processes of detachment. Not resting upon the juxtaposition of subjective emotions with distanced objectivity allows us to consider detachment relationally and on its own terms in ethnographic context (Trundle 2018; Yarrow *et al.* 2015). Detachment in this way is not primarily about being objective or emotionally distanced. As we have illustrated in shifting participant‐researcher relationships, and encountering collected data from a different vantage, detachment is about being *in relation to* engagement. Relational detachment may include spending time away from participants and the field, but may not depend on this. Through this lens, detachment is a pragmatic strategy employed at different points alongside engagement. This is an important part of managing ethical, personal, and practical tensions, whilst still being critically engaged with research.

The researcher does not have to be intensely emotionally engaged all the time to produce the kinds of inisghts that Rosaldo (2017), for example, describes. This is why the notion of ethnographic closeness is important. Ethnographic closeness denotes the methodological blend of engagement and detachment that must shift across the research process. Here, both engagement and detachment shape and are shaped by the ethnographer's own embodied relations to participants and their lifeworlds. Alongside the emotional, these relations involve moving in and out of the field, and attending to (shared) sensory experience. We frame the interplay of engagement and detachment as a duality to argue against mutual exclusivity, and we highlight how they are both grounded in and informed by the practical undertakings of ethnographic research to continuously shape each other in non‐linear ways. This interplay can culminate in unexpected encounters, engagements with data, and insights, even after leaving the field. Resulting from distancing practices, both the researcher and participants may re‐encounter and re‐engage with data, themes, and findings. As discussed, research relationships – even with those who have died – are never quite closed, as the researcher returns to materials collected and produced. Generating and maintaining appropriate levels of ethnographic closeness for the researcher is therefore a complex and ongoing negotiation of how to balance engagement and detachment, as they shift between more or less intimate and distant spaces, relationships, and research activities that intertwine.

I still reflect on my ongoing commitment to participants, as their lives come in and out of focus, and my memories of them are sometimes shadowed by the quiet question: *Are you still alive?* My emotions throughout this study have been complex, certainly not guilt‐free, but are merely one part of the intertwined, multifaceted picture of research experiences which seem to be somehow always unfinished. I reflect on creating dependence on my presence, refusing the offers to come for tea after the study was done, and all the times I was horrified at myself for being a day or two later than I intended when calling participants for a chat. Such are some of the emergent pressures and tensions of immersive research, where you become sensitized and sensitive to interlocutors’ needs, desires, and cares – blurring the ethical, the personal, and the anthropological. As ethnographers leaving the field may contend with a sense of abandonment or the absence of care for research participants (Biehl 2013), this intersection involving morality and emotions in ethnographic practice may be an important direction for future work.

Our depiction of ethnographic closeness responds to calls for transparency in ethnographic research methods and experiences in end‐of‐life research and anthropology more broadly. To continue to more fully furnish ethnographic toolkits and work to relieve some of the enormous pressures researchers may feel when conducting such projects, further contributions in this vein are needed. In pursuit of ethnographic closeness, engagement and detachment must be seen as complementary, and their nature should be explicitly expanded. We have illustrated some of these alternatives here. Presenting detachment as the methodological companion to engagement more readily speaks to the shifting positionalities, relationships, contexts, and research activities through which ethnographic closeness is generated and maintained in rigorous immersive research.

## Note on contributors

All authors collaborated to design the study, draft the study protocol, and secure study approvals. CP undertook primary data collection with support from TG and CMP as DPhil supervisors. Data analysis was conducted by CP, with input from TG and CMP to shape literature and theoretical engagement. CP wrote the first draft of the manuscript, with review and revision by all authors. TG secured funding for the study. All authors contributed to interpretation of study results and approved the manuscript for submission.

## NOTE

1 All names are pseudonyms.

## Appendix

**Table A1**. Exploratory phase (first 6 months).

**Table:**

**Activity**	**Date range**	**Visits**	**Total hours spent**
Care Certificate training course	12/2017	1 lasting 8 hours	8 hours
Life history interviews with 6 pilot participants	11/2017‐02/2018	6 lasting 2 hours	∼12 hours
Observations of HFSN*‐led heart failure clinics	10/2017‐02/2018	8 lasting 4 hours	∼32 hours
Observations of heart failure support groups	01/2018‐02/2018	2 lasting 3 hours	∼6 hours
Patient home visits with HFSNs*	10/2017‐02/2018	40 lasting 1‐2 hours	∼76 hours
**TOTAL ∼ 134 hours**			

*HFSN = Heart Failure Specialist Nurse.

**Table A2**. Ethnographic phase (latter 18 months).

**Table:**

**Participant**	**Date range of participation**	**Visits**	**Phone calls**	**Total hours spent**
Rosemary	22 months 02/2017‐12/2018	10 lasting 2‐3 hours	∼20 lasting ∼30 minutes	∼40 hours
Emily	24 months 02/2017‐02/2019	6 lasting 2‐3 hours	∼24 lasting ∼45 minutes	∼34 hours
Nick	3 months (deceased) 02/2017‐05/2017	1 with Nick 1 with GP 1 with family member	3 lasting ∼15 minutes	∼8 hours
Carol	2 months (discontinued) 09/2017‐10/2017	2 lasting 2 hours	3 lasting ∼15 minutes	∼5 hours
Theo	2 months (deceased) 11/2017‐01/2018	1 with Theo 1 with nurse	3 lasting ∼30 minutes	∼5 hours
Nancy	4 months (deceased) 02/2018‐06/2018	2 lasting 3 hours	∼8 lasting ∼30 minutes	∼10 hours
Noémie	12 months 02/2018‐02/2019	6 lasting 2‐3 hours	∼12 lasting ∼30 minutes	∼24 hours
Baylee	10 months 03/2018‐12/2018	5 lasting 2‐3 hours	∼10 lasting ∼30 minutes	∼20 hours
Philip	7 months 05/2018‐12/2018	4 lasting 2 hours	∼7 lasting ∼20 minutes	∼11 hours
Norma	7 months 06/2018‐01/2019	3 lasting 2 hours	∼7 lasting ∼30 minutes	∼12 hours
Kent	6 months 06/2018‐12/2018	4 lasting 2‐3 hours	∼8 lasting ∼30 minutes	∼15 hours
Jeanne	5 months (deceased) 06/2018‐10/2018	3 lasting 3 hours	6 lasting ∼10 minutes	∼10 hours
Kenneth	6 months 06/2018‐12/2018	3 lasting 2‐3 hours	6 lasting ∼30 minutes	∼10 hours
Edwin	6 months 06/2018‐12/2018	3 lasting 3 hours	6 lasting ∼30 minutes	∼12 hours
**TOTAL ∼216 hours**				
**TOTAL ∼350 hours over 24 months (2017‐19)**				
